# Methylation-associated Has-miR-9 deregulation in paclitaxel- resistant epithelial ovarian carcinoma

**DOI:** 10.1186/s12885-015-1509-1

**Published:** 2015-07-08

**Authors:** Xiao Li, Qianqian Pan, Xiaoyun Wan, Yuyan Mao, Weiguo Lu, Xing Xie, Xiaodong Cheng

**Affiliations:** 1 Women’s Reproductive Health Laboratory of Zhejiang Province, Women’s Hospital, School of Medicine, Zhejiang University, No.1 Xueshi Road, 310006 Hangzhou, Zhejiang China; 2 Department of Gynecologic Oncology, Women’s Hospital, School of Medicine, Zhejiang University, No.1 Xueshi Road, 310006 Hangzhou, Zhejiang China; 3 Zhejiang Financial College, No. 118 Xueyuan Street, 310018 Hangzhou, Zhejiang China

**Keywords:** Ovarian carcinoma, Chemoresistance, miR-9, Methylation

## Abstract

**Background:**

Drug resistance is still one of the key causes of death in epithelial ovarian carcinoma (EOC) patients, however there are very few strategies to reverse chemoresistance. Here we try to clarify whether and how miR-9 takes part in the regulation of paclitaxel sensitivity.

**Methods:**

miR-9 expressions in EOC cells and tissues were detected by Realtime PCR. The target of miR-9 was validated through dual luciferase reporter assay and Western Blot. Methylation study, RNAi technique and cytotoxicity assay were used to determine the intrinsic mechanism of miR-9 in paclitaxel sensitivity regulation.

**Results:**

miR-9 is down-regulated in paclitaxel resistant EOC. The patients with lower miR-9, Grade 3, Stage III –IV and suboptimal surgery present shorter survival time. miR-9 and suboptimal surgery are independent prognostic factors of EOC. Modulating miR-9 expression could change paclitaxel sensitivity of EOC cells. CCNG1, validated as a direct target of miR-9, mediates paclitaxel resistance. *miR-9-1* and *3* gene hypermethylation would decrease miR-9 expression, while demethylation of miR-9 gene could restore miR-9 expression and improve paclitaxel sensitivity in chemoresistance EOC cells. Furthermore, methylation-associated miR-9 deregulation in EOC cells could be induced by paclitaxel exposure.

**Conclusions:**

Methylation-associated miR-9 down-regulation is probably one of the key mechanisms for paclitaxel resistance in EOC cells, via targeting CCNG1. Our findings may also provide a new potential therapeutic target to reverse paclitaxel resistance in EOC patients.

**Electronic supplementary material:**

The online version of this article (doi:10.1186/s12885-015-1509-1) contains supplementary material, which is available to authorized users.

## Background

Although improved combination of surgery and chemotherapy is commonly applied for epithelial ovarian carcinoma (EOC), EOC is still the leading cause of death among gynecologic cancer nowadays [1, 2]. Initial response to chemotherapeutic drugs can be achieved in about 70 % EOC patients, but most of patients eventually develop chemoresistance and succumb to their diseases [1, 3]. Numerous evidences showed that chemoresistance is a clinically formidable problem in managing EOC patients. Obviously, reversion of drug resistance would contribute to improve prognosis.

As we know,miRNAs are small noncoding RNAs involved in the initiation and progression of human cancer [4]. Previous studies have suggested that miRNAs can act as oncogenes or tumor suppressors, exerting a key function in tumorigenesis and progression [5, 6]. For instance, miR-9 is under-expressed in gastric cancer, and ectopic expression of miR-9 can influence cell growth and cell cycle [7]. As a highly conserved miRNA found in insects and primates, miR-9 has three independent gene loci: *miR-9-1*, *miR-9-2*, and *miR-9-3*, located at chromosomes 1, 5, and 15, respectively [8]. miR-9 expression can be epigenetically modified. Studies showed that miR-9 deregulation and gene methylation was associated with cancer development and recurrence [9–11]. Heller [12] recently found that *miR-9-3* methylation was related to shorter overall survival and disease-free survival of lung squamous cell carcinoma patients. But no study, to our best knowledge, has been reported about the intrinsic relationship between miR-9 deregulation and paclitaxel resistance in cancer research up to today.

Our previous studies have identified a deregulated miRNA profile in paclitaxel resistant EOC using miRNA microarray and Realtime PCR [13]. Of those, miR-9 is one of the top down-regulated miRNAs, which implies that miR-9 might participate the regulation process of chemoresistance. In present study we try to inspect whether miR-9 take part in the process of chemoresistance regulation, and how about the methylation status of three miR-9 gene loci is in paclitaxel sensitive and resistant EOC. Which would help us to understand chemoresistant mechanism at the molecular level and illuminate fundamental properties of drug resistance in EOC.

## Methods

### Patient’s characteristics

In total 66 human epithelial ovarian carcinoma tissues were collected from Women’s Hospital, School of Medicine, Zhejiang University. All patients received chemotherapy including paclitaxel after primary surgery. Patients who had undergone preoperative radiotherapy or chemotherapy were excluded. All samples were immediately snap-frozen in liquid nitrogen and stored at −80 °C. Tumor histology was evaluated by an expert pathologist. Written informed consent was obtained from the participants and the study was approved by the ethical committee of Women’s Hospital, School of Medicine, Zhejiang University (Reference number: 20110027). The characteristics of the patients are listed in Additional file 1: Table S1. The term of paclitaxel resistant, paclitaxel sensitive, overall survival time (OS) and progression free survival time (PFS) was defined as before [13]. Since First-line treatment for EOC patients is usually based combined therapy, paclitaxel resistance is actually resistance to treatment (both paclitaxel and platinum). We will use chemoresistant or chemosensitive instead of paclitaxel resistant and sensitive for EOC patients.

### Cell culture and transfection

The EOC cell line SKOV3 was purchased from American Type Culture Collection (Manassas, VA, USA). Paclitaxel resistant cell line SKOV3-TR30 (ST30) was induced from SKOV3 [14]. The EOC cell line A2780 (European Collection of Cell Cultures, Salisbury, Wiltshire, UK) and its pacilitaxel resistant variants A2780R were obtained from professor Ding Ma (Tongji hospital, Tongji medical college, Huazhong university of science and technology, Wuhan, China).

Regulation of miR-9 was performed as before [13]. To regulate the expression of Cyclin G1 (CCNG1), cells were transfected with three different CCNG1 siRNA 1, 2, 3, or their negative control (50nM) (Ribobio, Guangzhou, China) by using Lipofectamine 2000(Invitrogen, Carlsbad, CA, USA). At 48 h after transfection, treated cells were harvested for reverse transcript-polymerase chain reaction (RT-PCR). To analyze the effect of miR-9 restoration upon demethylation, cells were seeded in six-well plates at a density of 1×10^6^ cells/ml and treated with 2uM 5-aza-2′-deoxycytidine (DAC, Sigma–Aldrich, St. Louis, MO, USA) for 72 h, replacing the drug and medium every 24 h.

### RNA extraction and realtime RT-PCR

Total RNA was extracted using TRIzol (Invitrogen) and RNeasy mini kit (Qiagen, Hilden, Germany) from ovarian cell lines or tissues. RNA concentrations were determined with Nanodrop 2000 thermo scientific spectrophotometer (Wilmington, DE, USA). RT reactions and Real-time PCR for miRNA and mRNA were performed as previously [13]. For miRNA quanitification, total RNA 0.5 μg (5ul), 62.5nM RT primer 1.0ul ((Ribobio) were incubated at 70 °C for 10 min and snapped on ice for 3 min, then added with 5 × RT Buffer 2.0 μl, dNTPs 0.5ul, RNase Inhibitor Protein 0.5ul, M-MLV 0.5ul (all from TaKaRa, DaLian, China) in a final volume of 10 μl, and incubated at 42 °C for 60 min, 70 °C for 15 min. Real-time PCR was performed using SYBR Premix Ex Taq kit (Takara, DRR081A). PCR volume was 20 μl, containing 1 μl RT product. Following cycling conditions were used [95 °C for 30 s, (95 °C for 5 s, 60 °C for 20 s, 70 °C for 10 s) × 40 cycles]. For mRNA, total cDNA was synthesized with the PrimeScript RT reagent Kit (TaKaRa, DRR037A) and Real-time PCR was performed using SYBR Premix Ex Taq kit (TaKaRa, DRR081A). The U6 snRNA and GAPDH were used as endogenous control for miRNA and mRNA respectively. The primers for CCNG1 and GAPDH were listed in Additional file 1: Table S2. Relative expression was calculated using the 2^−ΔΔCt^ method. ΔCt (miR-9) = Ct (miR-9)-Ct (U6), while ΔCt (CCNG1) = Ct (CCNG1)-Ct (GAPDH) in the same sample. ΔΔCt = (Group resistant ∆Ct) - (Group sensitive Group ∆Ct). Group ∆Ct was the ∆Ct mean of the paxlitaxel sensitive cells or tissues.

### Western blotting

Total protein extracts from the cells were prepared at 72 h after transfection. Western blot analysis was performed as previously [13], using the primary antibody against CCNG1 (sc-8016, 1:500, clone F-5, Santa Cruz, CA, USA), GAPDH (sc-25778, 1:1000, Santa Cruz) was used as an endogenous control.

### Cytotoxicity assay

To determine the effect of miR-9, DAC and CCNG1 siRNA2 on paclitaxel sensitivity of EOC cells, the cells treated with different conditions were suspended in 96-well plates (5 × 10^3^cells/well) overnight, then paclitaxel (Bristol-Myers Squibb, New York, NY, USA) was added in gradually increasing concentration (0, 1, 10, 50, 500, 1000nM) for 72 h. The cells exposed to culture medium only used as control. Viability of cells was determined using Cell-Titter 96 AQ_ueous_ One Solution Cell Proliferation Assay (MTS, Promega, Madison, WI, USA). In brief, 20 μL Reagent was added to each well, and incubated for 3 h. The absorbance was read on a Varioskan Flash spectral scanning Multimode Reader (Thermo Scientific) at 490 nm. Three wells were used for each condition, and experiments were performed in triplicate. The inhibited rate of EOC cells = 1 - the absorbance of EOC cells treated with paclitaxel/the absorbance of control EOC cells. IC50 values (the concentration of drugs that produced a 50 % reduction of absorbance) were analyzed.

### Dual luciferase reporter assay

Dual luciferase reporter assay was performed as previously [13]. In brief, the 3′-untranslated region (UTR) of CCNG1 (1367BP) mRNA containing the miR-9 binding site were PCR amplified, and cloned into the pmiR-RB-REPORT™ dual luciferase reporter vector (Promega). Site-directed Gene Mutagenesis Kit (Beyotime, Jiangsu, China) was used to produce the mutations of the miR-9 targeting site. The primers and mutation primers were synthesized by RiboBio and listed in Additional file 1: Table S2. The luciferase activities were measured at 48 h after cotransfection with miRNA mimic or its negtive control (50 nM) and different reporter vectors (50nM). The experiments were performed in triplicate and repeated three times.

### Methylation studies

To analyze the methylation status of miR-9 genes family (*miR-9-1*, *miR-9-2* and *miR-9-3*), bisulfite sequencing (BSP) and methylation-specific polymerase chain reaction (MSP) were carried out as described previously [10]. Genomic DNA was extracted from tissue sample and cell lines using AxyPrep Multisource Genomic DNA Minprep kit (Axygen, Hangzhou, China), and treated with sodium bisulfite using the EZ DNA Methylation-Gold kit (Zymo Research, Orange, CA, USA). For bisulfite sequencing, amplified PCR products were cloned into PMD18T vector (TAKARA), and 10–12 clones from each sample were sequenced.

### Statistical analysis

Kaplan-Meier survival functions and log-rank test were used to assess PFS and OS based on median miR-9 expression level. To further determine whether miR-9 is associated with survival, univariate and multivariate Cox Regression analysis were applied. Other data were analyzed using chi square test or student’s *t*-test. All statistical analyses were two-sided and performed with SPSS 11.5 software package. P-values less than 0.05 were considered statistically significant.

## Results

### miR-9 expression is down-regulated in paclitaxel resistant EOCs and correlated with prognosis

**Fig. 1:**
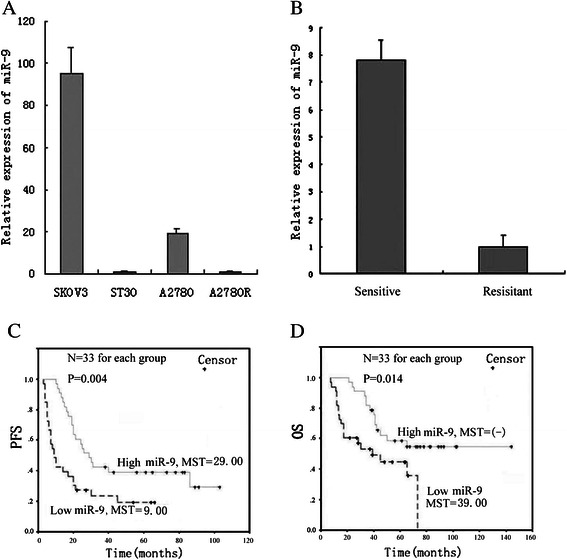
miR-9 expression in EOC and its clinical significances. **a**. Realtime RT-PCR for miR-9 in ovarian carcinoma cell lines (*P* = 0.000). **b**. Realtime RT-PCR for miR-9 in ovarian carcinoma tissues (*P* = 0.000). The experiments were performed in triplicate. **c**. Progression free survival time (PFS) of 66 EOC patients by miR-9 level (*P* = 0.004), MST, median PFS in months. **d**. Overall survival time (OS) of 66 EOC patients by miR-9 level (*P* = 0.014). MST, median OS in months

In accordance with our previous results [13], we validated that miR-9 expressions were reduced by 95.26-fold and 18.96-fold in paclitaxel resistant ST30 and A2780R cell lines, compared with their parental cell lines respectively (Fig. 1a). Further detection revealed that miR-9 expressions in 22 chemoresistant EOC patients were reduced by 7.80-fold compared with 44 chemosensitive EOC patients (Fig. 1b), which was consistent with our previous result of formalin-fixed paraffin-embedded samples [13]. In addition, we divided all tissues into high and low miR-9 expression group based on the median miR-9 value, and found a significantly longer progression free survival time (29.00(21.12-36.88) months) and overall survival time (not yet reached, as clearly showed by relative curve) in patients with higher miR-9 expression than those with lower miR-9 level (9.00(5.62-12.38) months and 39.00(14.53-63.47) months respectively), as shown in Figure 1c and d. Moreover, univariate cox analysis showed that lower miR-9, Grade 3, Stage III –IV and suboptimal surgery were associated with poor PFS (HR = 0.43, 0.34, 0.21 and 0.33 respectively) and OS (HR = 0.43, 0.26, 0.08 and 0.25 respectively). Type II cancer was associated with poor OS (HR = 0.07). Multivariate cox analysis revealed that lower miR-9 and suboptimal surgery were independent predictors for poor PFS (HR = 0.24 and 0.24) and OS (HR = 0.37 and 0.41) (Table 1). Hence, our data confirm that miR-9 expression is down-regulated in chemoresistant EOCs, and lower miR-9 predicts poorer prognosis of EOC patients.

**Table 1:** Univariate and multivariate Cox regression analysis of PFS and OS

	PFS		OS	
	HR(95 % CI)	*P* value	HR(95 % CI)	*P* value
Univariate analysis				
Age	0.98(0.95-1.01)	0.148	0.99(0.96-1.02)	0.481
miR-9(high vs low)	0.43(0.24-0.77)	0.005	0.43(0.21-0.86)	0.018
Grade1, 2 vs 3	0.34(0.17-0.67)	0.002	0.26(0.11-0.64)	0.003
Stage I-II vs III-IV	0.21(0.07-0.58)	0.003	0.08(0.01-0.61)	0.015
Optimal vs Suboptimal	0.33 (0.18-0.59)	0.000	0.25 (0.13-0.51)	0.000
Type I vs Type II	0.52(0.24-1.13)	0.099	0.07(0.01-0.51)	0.009
Multivariate analysis				
miR-9	0.24(0.12-0.50)	0.000	0.37(0.18-0.76)	0.007
Stage I-II vs III-IV	0.31(0.11-0.89)	0.029	0.15(0.02-1.16)	0.069
Optimal vs Suboptimal	0.24(0.11-0.53)	0.000	0.41(0.20-0.85)	0.016
Type I vs Type II	0.87(0.38-2.01)	0.750	0.11(0.01-0.78)	0.028

HR, hazard ratio; CI, confidence interval

### Modulating miR-9 expression changes paclitaxel sensitivity of EOC cells

**Fig. 2:**
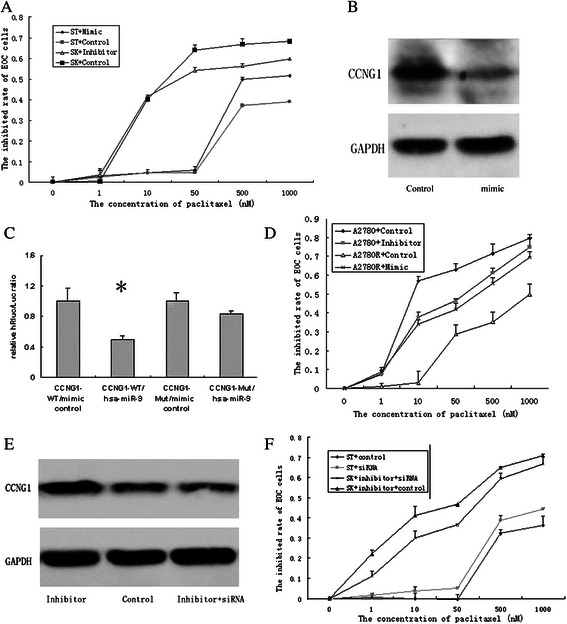
CCNG1 is the direct target of miR-9 and modulates the paclitaxel sensitivity of EOC cells **a**. Modulating miR-9 expression changed paclitaxel sensitivity of ST30 and SKOV3 cells. ST30 cells were transfected with miR-9 mimic or negative control(IC50 = 820.89 ± 21.62 nM VS 2424.56 ± 56.83nM, *P* = 0.001), SKOV3 cells were transfected with miR-9 inhibitor or negative control (IC50 = 122.74 ± 10.12 nM VS64.63 ± 2.74 nM, *P* = 0.000), the cytotoxicity of paclitaxel on EOC cells were assessed by MTS assay. **b**. Western blot analysis of CCNG1 in ST30 cells transfected with miR-9 mimic or negative control. GAPDH was used as house-keeping gene. **c**. Dual luciferase reporter assay. 293 T cells were transfected with CCNG1 -wild type 3′UTR vectors or mutant 3′UTR vectors together with miR-9 mimic or its negative control. Luciferase activity was measured 48 h after cotransfection. A decrease of the luciferase activity was observed in miR-9 overexpressing cells compared with control (* *P* = 0.008). **d**. Modulating miR-9 expression changed paclitaxel sensitivity of A2780 and A2780R cells. A2780 cells were transfected with miR-9 inhibitor or negative control (IC50 = 95.644 ± 12.03 nM VS 38.16 ± 6.18 nM, *P* = 0.000), A2780R cells were transfected with miR-9 mimic or negative control(IC50 = 194.94 ± 9.36 nM VS 774.03 ± 49.19 nM, *P* = 0.002). **e**. Western blot analysis of CCNG1 in SKOV3 cells transfected with miR-9 inhibitor, negative control or inhibitor combined with CCNG1 siRNA. GAPDH was used as house-keeping gene. **f**. Modulating CCNG1 expression changed paclitaxel sensitivity of ovarian carcinoma. Knockdown of CCNG1 alone enhanced paclitaxel cytotoxcity to ST30 cells (IC50 = 1468.50 ± 32.19 nM VS 2545.84 ± 168.83 nM, *P* = 0.000), while deleption CCNG1 reversed the role of miR-9 inhibitor on the paclitaxel sensitivity of SKOV3 cells (IC50 = 65.35 ± 13.47 nM VS 177.36 ± 20.88 nM, *P* = 0.001). The experiments were repeated three times

A significant reduction of miR-9 level was observed in SKOV3 cells after miR-9 inhibitor transfection, and a significant increase of miR-9 level was observed in ST30 cells after miR-9 mimic transfection (Additional file 2: Figure S1A, B). The cytotoxic effect of paclitaxel on EOC cell lines was assessed after transfection of miR-9 mimic or inhibitor (or negative control). miR-9 mimic induced a decreased IC50 value of paclitaxel in ST30 and A2780R cells, whereas miR-9 inhibitor brought an increased IC50 value in SKOV3 and A2780 cells (Fig. 2a, d). These data suggest that elevated miR-9 expression enhances paclitaxel cytotoxicity to drug-resistant EOC cells, while reduced miR-9 expression inhibits paclitaxel cytotoxicity to drug-sensitive EOC cells.

### CCNG1 is one of the targets directly regulated by miR-9

TargetScan database (www.targetscan.org, Released 6.0, Nov 2011) predicted CCNG1 contain the putative miR-9 binding site. Western blot validated that up-regulated miR-9 could inhibit CCNG1 expression in ST30 cells, while down-regulated miR-9 enhanced CCNG1 expression in SKOV3 cells (Fig. 2b, e). Using dual luciferase reporter assay, we found that the relative luciferase activities were significantly reduced in cells transfected with CCNG1 WT- 3′UTR/miR-9 mimic vectors compared with those transfected with CCNG1 WT-3′UTR/miR-9 mimic control (Fig. 2c). Furthermore, Realtime RT-PCR suggested that mRNA expression of CCNG1 in ST30 cells was significantly higher than that in SKOV3 (2.14 fold), which was contrary to the miR-9 expression trends in SKOV3 and ST30 cells (Additional file 2: Figure S1C). These data validate that miR-9 can directly bind to 3′UTR of CCNG1 and CCNG1 is regulated by miR-9.

### CCNG1 depletion enhances the paclitaxel sensitivity of EOC cells

CCNG1 was initially identified as a p53-regulated transcript induced by DNA damage [15]. Although its precise role on cellular growth control is still controversial, CCNG1 has been regarded as an oncogene [16, 17]^.^ CCNG1 gene copy number is an independent marker of postsurgical survival in EOC patients who have received chemotherapy with taxanes and platinum compounds [18]. Thus it suggests that CCNG1, the target of miR-9, probably modulates the paclitaxel-sensitivity of EOC. To validate this hypothesis, we knocked down CCNG1 in ST30 cells through transfecting CCNG1 siRNA. The roles of CCNG1 siRNAs were confirmed using realtime RT-PCR and Western blot, and the most effective siRNA was chosen (Additional file 2: Figure S1D, E). IC50 of paclitaxel in ST30 cells was significantly decreased after CCNG1 depletion (Fig. 2f), which confirmed that knockdown of CCNG1 alone would enhance paclitaxel cytotoxicity to EOC cells. Furthermore, when miR-9 inhibitor was transfected into SKOV3 cell accompanied with CCNG1 siRNA, the CCNG1 expression and the affection of miR-9 inhibitor on paclitaxel sensitivity were significantly reversed (Fig. 2e, f). Therefore, the results indicate that CCNG1, as one direct target of miR-9, participates in the regulation of paclitaxel-sensitivity in EOC cells.

### *miR-9-1* and *3* loci are hypermethylated in paclitaxel resistant EOC cells

**Fig. 3:**
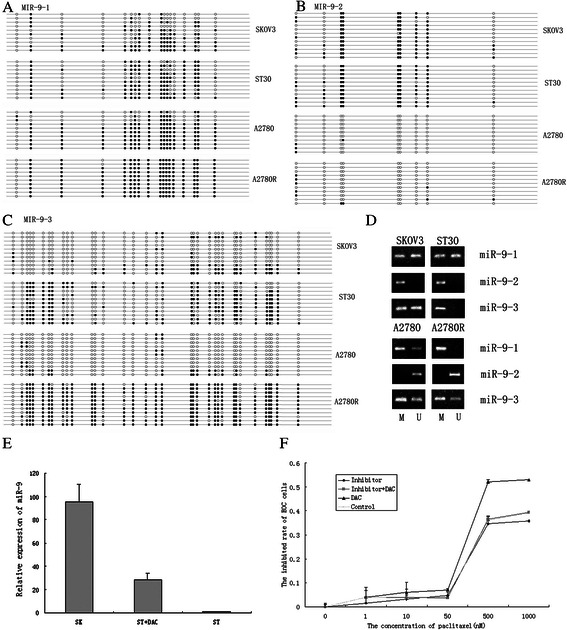
Methylation status of Hsa-miR-9 genes in EOC cells and their effects on the paclitaxel sensitivity. **a**. BSP results of the *hsa-miR-9-1* CpG island region in two pairs of paclitaxel sensitive and resistant EOC cell lines. 10 clones were sequenced for each cell line. Each circle indicates a CpG dinucleotide, black circle: methylated CpG; open circle: unmethylated CpG. **b**. BSP results of the *hsa-miR-9-2* CpG island region in two pairs of paclitaxel sensitive and resistant EOC cell lines. 10–12 clones were sequenced for each cell line. **c**. BSP restuls of the *hsa-miR-9-3* CpG island region in two pairs of paclitaxel sensitive and resistant EOC cell lines. 11 clones were sequenced for each cell line. **d**. MSP of three *hsa-miR-9* genes in different ovarian carcinoma cell lines. **e**. Real-time RT-PCR analysis of miR-9 in ST30 cell lines treated with or without DAC. Low miR-9 expression in ST30 cells was restored by DAC (*P* = 0.000). **f**. The effect of DAC and miR-9 inhibitor on the paclitaxel sensitivity of ST30 cell lines. The inhibited rates of paclitaxel on ST30 cell lines treated with or without miR-9 inhibitor, DAC combined with miR-9 inhibitor or DAC were assessed by MTS assay and the IC50 values were 3295.54 ± 154.87nM, 2590.36 ± 126.68nM, and 2057.35 ± 13.54nM in turn, compared with control (IC50 = 2898.94 ± 155.75 nM), *P =* 0.001, 0.056 and 0.035 in turn. The experiments were repeated three times

BSP revealed higher frequency of DNA methylation of *miR-9-1* and *miR-9-3* in ST30 and A2780R cells compared with their parental cells (Fig. 3a-c and Table 2). Again, MSP presented similar results (Fig. 3d). We further detected the methylation status of all three independent miR-9 gene loci in 66 EOC tissues using MSP and found chemo-resistant specific DNA hypermethylation patterns. The *miR-9-1* and *3* genes exhibited a significantly DNA hypermethylation status in the chemoresistant tissues compared with chemosensitive tissues, as shown in Table 3. Thus, our data suggest that decreased miR-9 expression might result from DNA hypermethylation in EOC cells.

**Table 2:** The methylation proportion of miR-9 genes in different EOC cell lines

	SKOV3	SK-treated	ST30	A2780	A2780-treated	A2780R
miR-9-1	48.89 %	64.44 %^#^	65.56 %^*^	78.33 %	92.22 %^#^	93.33 %^*^
miR-9-2	74.07 %	78.70 %	75.76 %	4.44 %	11.11 %	10.10 %
miR-9-3	21.56 %	28.29 %^##^	55.84 %^**^	11.43 %	18.86 %^##^	88.05 %^**^

The methylation proportions of miR-9-1 and miR-9-3 in ST30 and A2780 cells were significantly higher compared with their parent paclitaxel sensitive EOC cell lines SKOV3 and A2780, *P = 0.001 and 0.000, **P = 0.000 and 0.000. The methylation proportions of miR-9-1 and miR-9-3 were also increased significantly in paclitaxel treated SKOV3 and A2780 cells. ^#^*P* = 0.002 and 0.000, ^##^*P* = 0.035 and 0.005

**Table 3:** MSP of three miR-9 genes in paclitaxel sensitive and resistant ovarian carcinoma tissues

		M	M/U	U	Ratio	*P*-value
*miR-9-1* ^a^	sensitive	5	17	0	18.18 %	
	resistant	1	43	0	2.27 %	0.006
*miR-9-2* ^b^	sensitive	0	13	9	59.09 %	
	resistant	0	17	27	38.64 %	0.116
*miR-9-3* ^b^	sensitive	0	17	5	77.27 %	
	resistant	0	21	23	47.73 %	0.022

*M* methylated, *M/U* homozygously methylated, *U* unmethylated

^a^The methylation ratio of *miR-9-1* was calculated as methylated patients/all patients, because none was unmethylated

^b^The methylation ratio of *miR-9-2* and *3* was calculated as homozygously methylated patients/all patients

### Regulating miR-9 gene methylation changes chemo-sensitivity of ST30 cells

To confirm the influence of DNA methylation on miR-9 expression and chemo- sensitivity in EOC cells, miR-9 level of EOC cells were detected after cultured with or without DAC, an unspecific demethylation reagent. We found that low miR-9 expression in ST30 cells could be restored by genomic DNA hypomethylation (Fig. 3e). Therefore, Regulating DNA methylation would change the transcriptional activity of miR-9 in paclitaxel resistant EOC cells.

MTS assay showed that the IC50 value of ST30 was significantly increased in miR-9 inhibitor treated group and significantly decreased in DAC treated group, compared with that in miR-9 inhibitor control group. While DAC combined with miR-9 inhibitor group had no significantly effect on IC50 of ST30. (Fig.3f). These results suggest that hypermethylation of miR-9 genes, mainly *miR-9-1* and *miR-9-3*, would down-regulate miR-9 expression of EOC. Consequently, decreased miR-9 expression results in paclitaxel resistance of EOC cells. Thus, miR-9 is probably a potential therapeutic target and ablation of miR-9 hypermethylation status may partly reverse chemo-sensitivity in paclitaxel resistance EOC cells.

### Paclitaxel induces decreased miR-9 expression in EOC cells through influencing DNA methylation

**Fig. 4:**
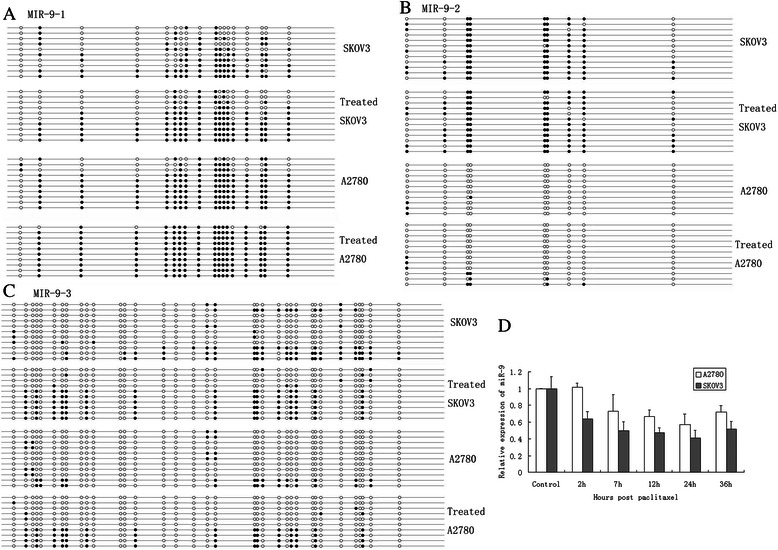
Paclitaxel down-regulates miR-9 expression in EOC cells through influencing DNA methylation. **a**. BSP results of the *hsa-miR-9-1* CpG island region in different ovarian carcinoma cell lines before and after 24 h paclitaxel treatment. 10 clones were sequenced for each cell line. Each circle indicates a CpG dinucleotide, black circle: methylated CpG; open circle: unmethylated CpG. **b**. BSP results of the *hsa-miR-9-2* CpG island region in different ovarian carcinoma cell lines before and after 24 h paclitaxel treatment. 10–12 clones were sequenced for each cell line. **c**. BSP results of the *hsa-miR-9-3* CpG island region in different ovarian carcinoma cell lines before and after 24 h paclitaxel treatment. 10–11 clones were sequenced for each cell line. **d**. Real-time RT-PCR analysis of miR-9 in SKOV3 and A2780 cell lines treated with paclitaxel at different time. The experiments were repeated three times

SKOV3 and A2780 were exposed to 10uM paclitaxel for 60 min before the drug was washed out. The miR-9 levels decreased after drug exposure in both cell lines, with the peak at 24 h (Fig. 4d), suggesting that the changes in miR-9 expression might be induced by paclitaxel. Since DNA hypermethylation was confirmed in paclitaxel resistant cells, we further determined whether the DNA methlyation status of ovarian carcinoma cell lines was changed after paclitaxel treatment. BSP results (Fig. 4a, c and Table 2) revealed a higher frequency of DNA methylation of *miR-9-1 and 3* in both cell lines at 24 h after drug exposure. In addition, DNA methylation of *miR-9-2* was also increased after drug exposure, although not significantly (Fig. 4b). Our findings suggest that paclitaxel resistance produced by methylation- associated miR-9 deregulation may be secondary from paclitaxel treatment in EOC cells.

## Discussion

Despite multiple new approaches of treatment, the high rates of death from EOC have remained largely unchanged for many years, with a five-year overall survival of only 30–39 % [19]. Dramatically poor prognosis of EOC patients is due to the development of chemoresistance. Therefore, it is imperative to identify new therapeutic targets that can reverse chemoresistance of EOC. miRNA represents a new class of small non-coding RNA that regulates multiple gene expression. Up-regulated miRNA could potentially target and down-regulate tumor suppressor genes, whereas down-regulated miRNA could potentially increase the expression of oncogenes [20]. Furthermore, studies suggested that miRNA might influence the response to chemotherapy [21–23]. Several studies have revealed that miR-9 possesses opposite functions in different types of cancer. For examples, miR-9 inhibits the growth of ovarian cancer and metastasis of gastric cancer [24, 25], and affects cell metabolism in cervical cancer [26]. Conversely, miR-9 promotes epithelial-mesenchymal transition and stimulates metastasis in breast cancer [27]. These opposing effects of miR-9 in different cancers imply that miR-9 is histological type specific and context-dependent. However, the effect of miR-9 on paclitaxel-sensitivity is still unclear up to date.

Paclitaxel is most widely used as a first-line therapeutic drug for EOC patients today. Here, we validated that miR-9 was significantly down-regulated in chemoresistant EOC cells and tissues, as our previous microRNA microarray result. miR-9 level was strongly correlated with PFS and OS of EOC patients and those with lower miR-9 expression presented poorer prognosis. The association of miR-9 level with prognosis implies a link between miR-9 and the paclitaxel-sensitivity of EOC. Just as we supposed, enforced expression of miR-9 significantly increased paclitaxel sensitivity in resistant EOC cells, while inhibition of miR-9 expression significantly decreased paclitaxel sensitivity in sensitive cells. Our findings suggest that miR-9 negatively regulates paclitaxel sensitivity and up-regulation of miR-9 probably can abolish paclitaxel-resistance of EOC cells.

Identification of miRNA gene targets helps us to understand the mechanism of miRNA function. According to the TargetScan database, we found over 1200 predicted Hsa-miR-9 targets, including some familiar oncogenes. Among them, CCNG1 is confirmed as one of the direct targets for miR-9, which is consistent with recent study [28]. Increased CCNG1 is accompanied with paclitaxel-induced spindle assembly checkpoint-mediated mitotic arrest and promotes cell survival after paclitaxel exposure [18]. Thus, CCNG1 may serve as a marker for the sensitivity of cancers to anti-mitotic therapy through regulating the outcome of taxane-induced mitotic arrest. Here, we found that depletion of CCNG1 increased the paclitaxel toxicity to EOC cells, and the effect of miR-9 inhibitor on paclitaxel sensitivity of SKOV3 was remarkably reversed by CCNG1 siRNA. Our findings indicate that CCNG1-mediated paclitaxel resistance might be induced by decreased miR-9. And miR-9, as well as its target gene CCNG1, may be one of the key pathways in regulating paclitaxel-sensitivity in EOC cells.

To identify epigenetic mechanism involved in aberrant miR-9 expression, methylation status of miR-9 genes in EOC was detected. We found methylations of the *miR-9-1* and *3* genes were significantly higher in paclitaxel resistant EOC than those in paclitaxel sensitive cells. Furthermore, demethylation reagent DAC not only restored miR-9 expression, but also enhanced the paclitaxel-sensitivity in resistant cells, suggesting that decreased miR-9 level in chemoresistant EOC results from DNA hypermethylation. As we know, chemoresistance can be de novo or acquired in clinical settings and may be a strategy by which cells stand against paclitaxel exposure [29]. To uncover the causation of miR-9 deregulation and DNA methylation in paclitaxel resistant EOC cells, we treated EOC cells with paclitaxel. Results revealed that DNA methylation of miR-9 was increased and miR-9 expression was decreased in EOC cells after 24 h paclitaxel expoure. These data suggest paclitaxel induces miR-9 gene hypermethylation and down-regulates miR-9 expression in EOC cells. Consequently, miR-9 down-regulation induces the CCNG1 overexpression and causes paclitaxel resistance. Thus, paclitaxel resistance in EOC cells may be secondary and result from paclitaxel treatment, which inversely results in chemotherapy failure. These findings not only help us to uncover an intrinsic pathway of paclitaxel-induced miR-9 down-regulation and CCNG1 overexpression, but also provide a novel insight into the underlying molecular mechanisms in chemoresistant EOC.

## Conclusions

In summary, we identified miR-9 as a regulator for paclitaxel sensitivity in EOC. miR-9 expression is regulated by gene hyperemthylation and related to prognosis. Demethylation of miR-9 gene would restore miR-9 expression and improve the paclitaxel sensitivity of EOC. Inversely, methylation-associated miR-9 deregulation could be induced by paclitaxel exposure in EOC cells. CCNG1, as a direct target of miR-9, could mediate paclitaxel resistance. These findings might provide a new potential therapeutic target to reverse paclitaxel resistance in EOC patients.

## Additional files

Additional file 1: Table S1. The characteristics of ovarian carcinoma patients. Table S2. The sequences of primers in Real time RT-PCR and the dual luciferase reporter assay.
Additional file 2: Figure S1. The trasnsfection efficiency in EOC cells validated by Real-time RT-PCR and Western Blot. A and B. Real-time RT-PCR analysis of miR-9 in transfected cells. Compared with negative control, (A) miR-9 mimic transfected ST30 cells showed a 2219.37 fold increase of miR-9 (*P* = 0.000), (B) miR-9 inhibitor transfected SKOV3 cells led to a 12.59 fold reduction of miR-9 compared with negative control (*P* = 0.002). C. Real-time RT-PCR analysis of CCNG1 in ST30 cell and SKOV3 cells. D. Real-time RT-PCR analysis of CCNG1 in ST30 cell lines treated with CCNG1 siRNA1, 2, 3 or their negative control. All three siRNA achieved more than 80 % interference efficiency. E. Western Blot analysis of CCNG1 in ST30 cell lines treated with CCNG1 siRNA1, 2, 3 or their negative control. siRNA2 was validated as the most effective siRNA.

## Abbreviations

EOC: Epithelial ovarian carcinoma
OS: Overall survival time
PFS: Progression free survival time
ST30: SKOV3-TR30
CCNG1: Cyclin G1
RT-PCR: Reverse transcript-polymerase chain reaction
DAC: 5-aza-2′-deoxycytidine
MTS: Cell-Titter 96 AQ_ueous_ one solution cell proliferation assay
IC50 values: The concentration of drugs that produced a 50 % reduction of absorbance
BSP: Bisulfite sequencing
MSP: Methylation-specific polymerase chain reaction
