# Supplementary material for: Methylation-associated Has-miR-9 deregulation in paclitaxel- resistant epithelial ovarian carcinoma
Source: BMC Cancer. 2015 Jul 8;15:509. doi: 10.1186/s12885-015-1509-1 (PMC4495847; doi:10.1186/s12885-015-1509-1)
Supplement: Additional file 1: Table S1. — The characteristics of ovarian carcinoma patients. Table S2. The sequences of primers in Real time RT-PCR and the dual luciferase reporter assay. [file 12885_2015_1509_MOESM1_ESM.doc]

**Table S1.** The characteristics of ovarian carcinoma patients

|  | Sensitive (N) | Resistant (N) |
| --- | --- | --- |
| Case | 44 | 22 |
| Year of age  Median (Range) | 49(31-89) | 50(32-68) |
| FIGO Stage |  |  |
| I | 9 | 0 |
| II | 4 | 1 |
| III | 31 | 19 |
| IV | 0 | 2 |
| Tumor Grade |  |  |
| 1 | 6 | 2 |
| 2 | 13 | 2 |
| 3 | 25 | 18 |
| Histology subtype |  |  |
| Serous carcinoma | 31 | 22 |
| Mucinous carcinoma | 5 | 0 |
| Endometrioid carcinoma | 3 | 0 |
| Clear cell carcinoma | 5 | 0 |
| Tumor type |  |  |
| Type I | 12 | 2 |
| Type II | 32 | 20 |
| Primary surgery |  |  |
| Optimal | 33 | 10 |
| Suboptimal | 11 | 12 |
| Recurrence |  |  |
| No  Yes | 19  25 | 0  22 |
| Survival |  |  |
| No  Yes | 13  31 | 20  2 |

**Table S2. The sequences of primers in Real time RT-PCR and the dual luciferase reporter assay**

| Primer | Sequence 5'-3' |
| --- | --- |
| CCNG1-F | GTGGTATTGAAAATGCTATTGGAGGA |
| CCNG1-r | ATAATGACAGTGATTGAAGCTGTGGG |
| GAPDH-F | GACAGTCAGCCGCATCTTCT |
| GAPDH-R | TTAAAAGCAGCCCTGGTGAC |
| CCNG1-WT-F | CCGCTCGAGCTGGATTATTACAGCACCAAAAAACTTCTCTG |
| CCNG1-WT-R | GAAT GCGGCCGCTTGAATTTCATTAACTTTATTGAAAGGTATATCC |
| CCNG1- Mut -F | ATGAAAA**TCGATACT**ACCACAGTATATCTTATTCT |
| CCNG1- Mut -R | GTAGTATCGATTTTCATTGTGTACTGATCCATAAC |

WT: wild type; F: forward, R: reverse; Mut: mutation. The mutation site in the Mut-primer is bold.
