# Supplementary figures and images for: Methylation-associated Has-miR-9 deregulation in paclitaxel- resistant epithelial ovarian carcinoma
Source: BMC Cancer. 2015 Jul 8;15:509. doi: 10.1186/s12885-015-1509-1 (PMC4495847; doi:10.1186/s12885-015-1509-1)

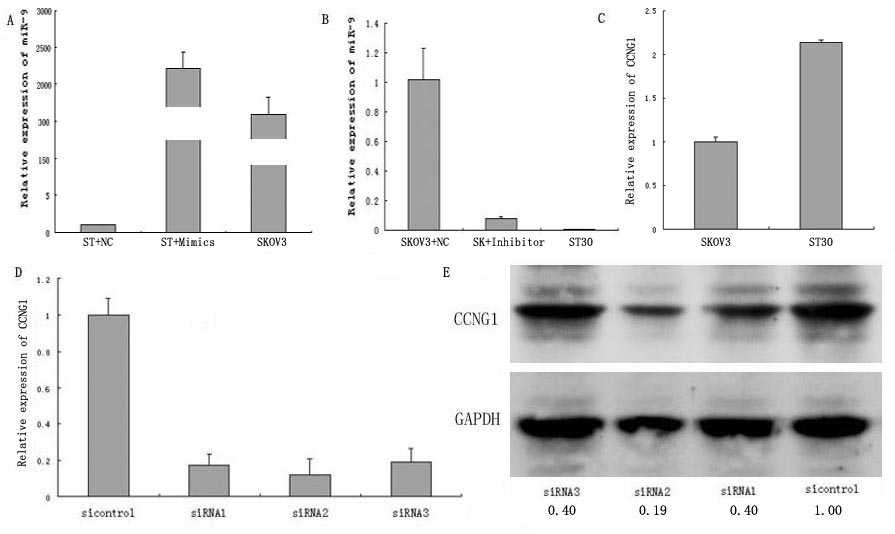

Supplement: Additional file 2: Figure S1. — The trasnsfection efficiency in EOC cells validated by Real-time RT-PCR and Western Blot. A and B. Real-time RT-PCR analysis of miR-9 in transfected cells. Compared with negative control, (A) miR-9 mimic transfected ST30 cells showed a 2219.37 fold increase of miR-9 (P = 0.000), (B) miR-9 inhibitor transfected SKOV3 cells led to a 12.59 fold reduction of miR-9 compared with negative control (P = 0.002). C. Real-time RT-PCR analysis of CCNG1 in ST30 cell and SKOV3 cells. D. Real-time RT-PCR analysis of CCNG1 in ST30 cell lines treated with CCNG1 siRNA1, 2, 3 or their negative control. All three siRNA achieved more than 80 % interference efficiency. E. Western Blot analysis of CCNG1 in ST30 cell lines treated with CCNG1 siRNA1, 2, 3 or their negative control. siRNA2 was validated as the most effective siRNA. [file 12885_2015_1509_MOESM2_ESM.jpeg]
